# Real-time PCR for diagnosing and monitoring treatment effect of *Strongyloides stercoralis* infection in a non-endemic setting

**DOI:** 10.3389/fpara.2023.1277372

**Published:** 2023-10-27

**Authors:** Linda J. Wammes, Suzanne A. V. van Asten, Lisette van Lieshout, Els Wessels, Jaco J. Verweij

**Affiliations:** ^1^Department of Medical Microbiology, LUMC Center for Infectious Diseases (LU-CID), Leiden University Medical Center, Leiden, Netherlands; ^2^Department of Parasitology, LUMC Center for Infectious Diseases (LU-CID), Leiden University Medical Center, Leiden, Netherlands; ^3^Microvida, Laboratory for Medical Microbiology and Immunology, Elisabeth-TweeSteden Hospital, Tilburg, Netherlands

**Keywords:** real-time (q)PCR, *Strongyloides stercoralis* (S. stercoralis), molecular diagnosis and detection, strongyloidiasis, strongyloidiasis follow-up

## Introduction

Strongyloidiasis is caused by the soil-transmitted helminth *Strongyloides stercoralis* and is potentially life threatening, especially in immunocompromised patients (Greaves et al., 2013). In spite of the long-term serious complications of undiagnosed chronic cases and the significant global burden, diagnosis of human strongyloidiasis remains challenging (Costa et al., 2021; Buonfrate et al., 2022). Over the past decades the laboratory diagnosis of strongyloidiasis has been primarily based on the detection of larvae by microscopic examination of stool samples. However, insufficient sensitivity of microscopy has been reported (Campo Polanco et al., 2014) and concentration techniques are cumbersome with only slight improvement in accuracy (Intapan et al., 2005). Certain serological tests demonstrate good sensitivity, but this is highly dependent on the type of test used. Moreover, because of possible cross reactions with antibodies to other parasites and long-term persistence of antibodies, the specificity remains suboptimal (Bisoffi et al., 2014; Swan et al., 2022). The diagnostic challenges in strongyloidiasis were also illustrated by the report of an 18-year cohort in London, in which 413 returning travelers and migrants treated for *S. stercoralis* were evaluated (Ming et al., 2019). A case of strongyloidiasis was defined as proven (confirmed through microscopy and/or culture) or presumed (positive serological test, without previous treatment). 86 patients (21.0%) had a proven diagnosis of strongyloidiasis based on positive stool microscopy or culture. Of this group, just more than half (54.7%) of the subjects were symptomatic and almost a quarter (23%) of them did not have peripheral blood eosinophilia. Serology was positive in 70/86 patients (sensitivity 81%), with a much lower sensitivity in travelers (46%). This report again emphasizes the absence of a gold standard test for detection of active and current *S. stercoralis* infection.

Molecular methods have been used in different countries and report variable accuracy depending on the reference technique and setting of the study (Verweij et al., 2009a; Knopp et al., 2014; Buonfrate et al., 2022). A meta-analysis including 14 studies of which some evaluated more than a single molecular method on the same group of patients or a single molecular method on different subsets of patients, leading to multiple estimates for test performance (Buonfrate et al., 2018). The comparison of all molecular techniques to conventional parasitological methods resulted in a sensitivity of 61.8% (95% CI:42.0 to 78.4) and a specificity of 95.2% (95% CI:92.0 to 97.2), increasing when studies with serology-positive patients were excluded.

The most commonly used real-time PCR (Buonfrate et al., 2022), targeting the ribosomal small subunit sequence (SSU rRNA), was developed at the Department of Parasitology of the Leiden University Medical Center (LUMC), the Netherlands (Verweij et al., 2009a). With high specificity and a two-fold increase in detection rate compared to the commonly used Baermann sedimentation method, this assay showed promise as a diagnostic tool (Verweij et al., 2009a; Meurs et al., 2017). Subsequently the test was implemented in the clinical microbiology laboratory of the LUMC (2005) and that of the Elisabeth-TweeSteden Hospital (ETZ) in Tilburg (2014). In the present study we aim to evaluate the performance of this *S. stercoralis* real-time PCR as a routine diagnostic test in two clinical microbiology laboratories situated in a non-endemic setting, including its usefulness for monitoring drug treatment.

## Methods

The present study is a retrospective analysis of diagnostic data collected over a period of more than 15 years in two clinical microbiology laboratories in the Netherlands, a tertiary referral laboratory situated in Leiden (LUMC: hospital A) and a teaching hospital situated in Tilburg (ETZ: hospital B). In hospital A, *S. stercoralis* PCR could be specifically requested not only by hospital physicians, but also by other clinical microbiology laboratories or treating physicians throughout the country when strongyloidiasis was suspected. In hospital B, stool samples submitted by regional general practitioners or hospital physicians were routinely screened for *S. stercoralis* by PCR when diagnosis of worm infections was requested without further clinical specifications.

In both hospitals, all diagnostic assays (PCR, microscopy and antibody testing) have been performed under current quality system requirements (ISO-15189:2012 accreditation or previously the national quality system CCKL), including successful participation in external quality assessment schemes.

### Data collection and ethical considerations

The laboratory information management systems (LIMS) of the two laboratories were searched for *S. stercoralis* PCRs that were performed during the period August 25^th^ 2005 until December 31^st^ 2022. Besides stool samples, other clinical materials were also included if tested by PCR. Where available, serology and microscopy results were extracted from patients with a positive PCR result. Corresponding clinical data, concerning for example geographical exposures and timing of treatment, were retrieved from enclosed information with the requested tests and from retrospective analysis of telephone logs when informing clinical microbiologists or treating physicians about the diagnostic results inside or outside the hospitals. Because of the retrospective nature of this study, without any additional intervention or use of biomaterials, no informed consent was obtained.

### Real-time PCR

#### Hospital A

Until January 2014 pretreatment and DNA extraction of feces samples was performed as described in van Maarseveen et al., 2010 (van Maarseveen et al., 2010). In January 2014 the pretreatment and DNA extraction method for feces was changed. Since then, 100-150 mg feces was transferred to a 2 mL tube containing Precellys Soil grinding SK38 beads (Bertin instruments, Montigny-le-Bretonneux, France) and 1.25 mL STAR buffer (Roche Diagnostics, Rotkreuz, Switzerland). The tubes were mixed in the Precellys 24 tissue homogenizer (Bertin instruments) on 5500 rpm for 10 s, followed by 60 s incubation and again 10 s 5500 rpm, followed by 2 minutes incubation in the machine. The tubes were incubated 5 minutes at room temperature and centrifuged for 60 s at 14000 rpm in an Eppendorf centrifuge. A 200 µL sample was transferred to a MagNA Pure LC or 96 cartridge and nucleic acids were extracted on the MagNA Pure LC or 96 instrument (Roche Diagnostics) using the MagNA Pure 96 DNA and Viral NA small volume kit. The *Strongyloides*-specific primers and probe targeting the 18S rRNA that were used for the real-time PCR were described in a previous paper (Verweij et al., 2009b). The PCRs were performed in a volume of 50 μL (until 2014) or 25 µl using HotStar Taq Master Mix (QIAGEN, Hilden, Germany), 5 mM MgCl_2_, 2.5 µg Bovine Serum Albumin (BSA) (Roche Diagnostics, Almere, The Netherlands), and optimized primer and probe concentrations. Amplification consisted of 15 min at 95°C followed by 45 cycles of 30 s at 95°C, 30 s at 55°C, 30 s at 72°C (until 2017) or 45 cycles of 5 s at 95°C, 15 s at 55°C, 15 s at 72°C and was performed using IQ5 or CFX96 Real-Time Detection Systems (Bio-Rad).

#### Hospital B

For DNA extraction, approximately 100 mg unpreserved feces was suspended in PBS with 2% polyvinypolypyrolidone (PVPP), followed by mechanical disruption through bead-beating 30 seconds at 3000 rpm (MagNa Lyser Green Beads, Roche), freezing and overnight lysis in sodium dodecylsulphate-proteinase K at 55°C. DNA was extracted using the QIAsymphony DSP virus/pathogen midi kit and pathogen complex 400 protocol of the QIAsymphony Sample Processing (SP) system (Qiagen). The *S. stercoralis* PCR was performed as part of two multiplex helminth PCRs targeting nine other helminths as well using primers and probes.

Amplification reactions were performed in a volume of 25μL with PCR buffer (QuantiTect Multiplex PCR NoROX Kit, QIAgen), 2.5μg BSA (Roche Diagnostics), and 10μL DNA sample. Amplification consisted of 15min at 95°C followed by 45 cycles of 15 s at 95°C, 30 s at 60°C, and 30 s at 72°C. Amplification, detection, and analysis were performed with the Rotor-gene real-time detection system (Qiagen).

In both centers a fixed amount of Phocine Herpes virus 1 (PhHV-1) was added to each sample, to serve as an internal control for the DNA extraction procedure and to monitor inhibition of the real-time PCR. Both laboratories have shown adequate scores in the Helminth External Molecular Quality Assessment Scheme (HEMQAS), offered since 2019 (Cools et al., 2020).

### Microscopy

For a subset of patients, data on microscopic examination were available. For microscopy, fecal samples were collected without the addition of preservatives. Routinely a formal-ether concentration procedure was performed according to Ridley, after which the sediment was screened for the presence of eggs, larvae and protozoa cysts (Brienen et al., 2017). The Baermann procedure was only performed when strongyloidiasis was suspected, the amount of feces was sufficient and the time gap between defecation and examination was less than 24 hours. For the Baermann method, fecal material was placed on a layer of 2 hydrophilic gauze bandages. The gauze was folded into a pouch and placed in a 50ml tube filled with bottled water for 3hrs. Following decantation, the remaining sediment was examined for nematode larvae (Brienen et al., 2017; Gelaye et al., 2021).

### Serology

On request, detection of *Strongyloides*-specific IgG (IgG1 and IgG4) antibodies in serum was performed by an in-house enzyme-linked immunosorbent assay (ELISA) (Polderman et al., 1994). This ELISA test has been implemented at the LUMC several decades ago as routine diagnostic test for the serological diagnosis of strongyloidiasis. For this assay, quality was externally monitored by successful participation in the UK NEQAS quality assessment scheme for parasite serology (Collier et al., 2010).

In brief, 96-well plates were coated with a homogenate of L3 stage larvae of *S. stercoralis* (obtained from the Laboratory of Parasitology; University of Pennsylvania School of Veterinary Medicine, Philadelphia, USA). After adding serum samples, reactive IgG1/IgG4 antibodies were detected by subsequently adding and washing away the following reagents: mouse anti-human IgG1/IgG4 (Sigma-Aldrich, Merck, Darmstadt, Germany), goat-anti-mouse IgG conjugated to alkaline phosphatase (Sigma-Aldrich), and 4-nitrophenyl phosphate substrate (Sigma-Aldrich).

On each plate a reference standard was included using a positive serum to determine the cut-off optical density. A negative and a positive control were assessed in each test run and used as acceptation criteria. The titer was determined as the dilution of the sample at which the extinction was higher or equal to the cut-off of the reference standard. Samples were considered negative showing a titer <1:40, weakly positive showing a titer of 1:40 or 1:80 and positive when showing a titer >1:80.

To assess concordant antibody response data next to PCR diagnosis, corresponding ELISA data were analyzed from ± 6 weeks around the date of feces PCR positivity.

### Follow-up data

For a subset of patients multiple samples were available. Since we did not have access to prescription data, we assumed treatment administration based on oral information from the consulting clinical microbiologists or treating physicians. To assess the clinical utility of the PCR for monitoring treatment effect, these data (Cq-value and date of testing) were also collected. The time to follow-up was calculated in days.

### Statistical analysis

All data were combined in SPSS Statistics (IBM, New York, USA); graphs were created in GraphPad Prism (GraphPad, Boston, USA). The descriptive nature of this study did not allow many statistical possibilities.

## Results

### Description of available samples and study participants

A total of 8116 PCR results were available in hospital A; the real-time PCR assay tested positive in 137 specimens from 92 patients with a median Cq value of 28.8 (range 15.8-40.0) in the primary feces sample. In hospital B, 11063 PCR results were obtained; real-time PCR tested positive in twelve specimens that were collected from eleven patients (median Cq value 26.7, range 14.8-35.1). Characteristics of the 103 patients with positive PCR results are summarized in **Table 1**. Faecal specimens were used as the primary specimen; for two patients there was only a positive PCR on a non-faecal specimen available (n=1 sputum, n=1 stomach biopsy). These samples were not included in follow-up analysis (see below). Other PCR-positive specimens during the study period included sputum, bronchoalveolar lavage fluid, cerebral spinal fluid (CSF), and skin or other tissue biopsies. Microscopy results were available for 36 of 103 patients (35%) and were negative in 17 (47%; **Table 1**). IgG1/IgG4 antibodies to *S. stercoralis* antigen were analyzed in 74 of 103 *Strongyloides* PCR-positive patients (78.1%). In almost all cases (70 of 74, 94.6%) antibodies could be detected. When limiting the analysis to those with serum samples collected within a timeframe of 6 weeks around the primary PCR-positive specimen, 63 patients were serologically tested of which 61 were antibody-positive (96.8%).

**Table 1:** Characteristics of patients with a positive *S. stercoralis* PCR result.

	Hospital A (n=92)	Hospital B (n=11)
**Gender, n male (%)**	71 (77)	7 (64)
**Median age in years (IQR)**	62 (19)	54 (25)
Reason for PCR, n (%)		
** GI symptoms** ** Eosinophilia** ** Eosinophilia & GI symptoms** ** Eosinophilia & other symptom(s)** ** Skin symptoms** ** Respiratory symptoms**	8 (9) 23 (25) 8 (9) 4 (4) 1 (1) 4 (4)	3 (27) 1 (9) 1 (9) 1 (9) 1 (9) 1 (9)
** Confirmation of microscopy**	6 (7)	
** Positive serology** ** Laesions or helminths found at scopy/histology** ** Screening before immune suppression** Other* ** Unknown**	8 (9) 7 (8) 3 (3) 3 (3) 17 (19)	3 (27)
Link to geographical area, n (%)		
** Asia** ** South East Asia** ** Surinam** ** Sub-Saharan Africa** ** Europe** ** Other/several endemic areas** ** Unknown**	2 (2) 13 (14) 20 (22) 8 (9) 3 (3) 8 (9) 38 (41)	1 (9) 1 (9) 1 (9) 2 (18) 0 0 6 (55)
**Microscopy, n performed (% of total)** ** Positive, n (% of performed)** ** Negative, n (% of performed)**	31 (34) 18 (58) 13 (42)	5 (46) 1 (20) 4 (80)
**IgG1&IgG4 serology, n performed (% of total)** ** Positive (% of performed)** Weak positive** (% of performed) ** Negative (% of performed)**	72 (78) 64 (88) 4 (6) 4 (6)	2 (18) 2 (100) 0 0

IQR interquartile range; GI gastrointestinal.

*Other reasons for diagnostics, for example anemia, headache, combinations of non-specific symptoms.

**Weak positive was defined as a titer of 1:40 or 1:80 dilution in the IgG1/IgG4 ELISA (cut-off 1:40).

### Follow-up diagnostics of *Strongyloides* PCR-positive patients

**Figure 1:**
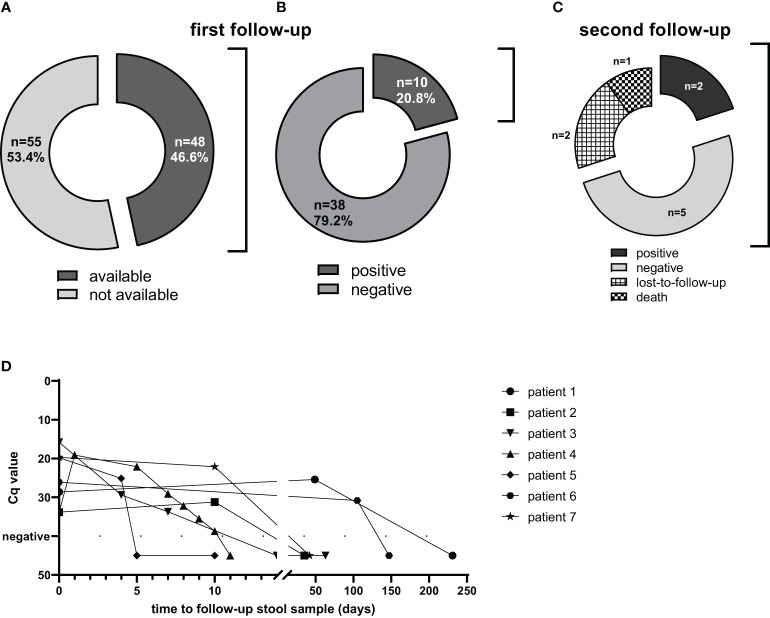
Use of PCR for follow-up after presumed treatment. From PCR-positive subjects (n=103), 48 follow-up samples were available **(A)**. From those, 38 samples were negative **(B)**. From the 10 subjects with a positive follow-up sample, 1 died, 2 were lost to follow-up and from 7 subjects further follow-up samples were obtained **(C)**. These seven patients were followed-up until negative **(D)**.

Next, PCR data were analyzed in follow-up samples of positive patients (**Figure 1**). Diagnostic follow-up of infected patients was not a common practice during the study period. PCR results on consecutive feces samples were available for 46.6% of the study population (**Figure 1A**). In 38 of those 48 (79%), the first follow-up feces sample was PCR negative (**Figure 1B**). For the remaining 10 patients, at least one positive follow-up sample was obtained (**Figure 1B**). One patient with disseminated strongyloidiasis was positive in one follow-up feces sample, thereafter persistently positive in sputum and passed away despite intermittent ivermectin treatment (van Westerloo et al., 2014). Two patients were lost-to-follow-up after the first positive follow-up sample, since they were seen in another hospital and no follow-up faecal samples were sent in. For five of the remaining seven subjects, the second follow-up sample was negative (**Figures 1C, D**). Two patients repeatedly tested positive and became negative at the third or seventh follow-up sample. Follow-up was, however, more frequent in these two individuals, who were negative in feces after 14 and 7 days respectively (**Figure 1D**).

The time between primary and follow-up fecal samples varied considerably (for the whole subgroup with follow-up sample(s): median 37 days, range 1 – 996 days). The elapsed time in the subgroup with a negative result of the first follow-up sample was similar (median 38.5 days). In 34 of 48 of the followed subjects (71%) a consecutive sample was obtained within eight weeks; 27 of those were directly negative (median time to negative 28 days, range 2 to 56 days).

## Discussion

In the present study we share more than 15 years of experience with an in-house real time PCR to detect *S. stercoralis*-specific DNA in a non-endemic setting. The PCR proved useful in detecting and following up infections and moreover detected additional cases compared to microscopy or serodiagnosis in a non-biased setting.

Due to the setting and the retrospective nature of the study it was not possible to directly compare the different diagnostic methods. The finding that a positive PCR was microscopically confirmed in only 53% of fecal samples, reflects the well-known suboptimal sensitivity of microscopy (Buonfrate et al., 2022). During the study period, no additional patients were identified by microscopy, while missed by PCR. It should be taken into account, however, that the Baermann method, which is considered to be the most sensitive concentration procedure for the detection of *S. stercoralis* larvae, was not applied to all stool samples. Also copro-culture methods that are known to provide a considerable increase in sensitivity are not routine practice in our laboratories. Although all traditional methods gain sensitivity by testing multiple samples, infections can also be missed by PCR on a single stool sample, as shown in a previous meta-analysis, although several DNA targets and different comparators were used (Buonfrate et al., 2018). In spite of its disadvantages, *Strongyloides* PCR has been regarded useful as complementary or confirming test in settings where immunocompromised patients are screened or where there are challenges to maintain microscopic competence (Costa et al., 2021; Buonfrate et al., 2022).

Even by serology, generally seen as a sensitive though less specific, method for diagnosing *S. stercoralis* infections, four PCR positive patients were missed. A possible explanation could be late seroconversion, but this could not be confirmed due to missing information on the time period between exposure and testing and absence of additional serum samples. Based on these and other reports, and our own experiences, we advise a combination of PCR and antibody testing to reach optimal sensitivity to detect infections.

The usefulness of *Strongyloides* PCR to monitor treatment efficacy has not been widely reported. It is known that after successful treatment antibody levels slowly decrease over time. In general it takes at least 6 to 12 months for patients to become sero-negative (Page et al., 2006), often depending on the test characteristics and the patient’s immune status (Buonfrate et al., 2022; Swan et al., 2022). This illustrates the need for a more accurate diagnostic test which can be used to monitor patients after the start of therapy. In the present cohort, most patients were PCR negative in a follow-up stool sample collected within eight weeks in some cases even within one week after ivermectin treatment. This was calculated from the sample date of first positive PCR result, so this includes the time of testing, reporting and the time to administration of treatment. If this finding is extrapolated to the clinical problem of imported strongyloidiasis, it would mean that the majority of infected patients would be discharged from follow-up after providing one (negative) follow-up stool sample within two months, instead of one year by using serology. In a setting where (multiplex) PCR testing is already incorporated in the daily work flow, this would mean a large increase in efficiency of care for these patients. Since the present study had a retrospective design, without per-protocol collection of follow-up samples, the suggested time period for follow-up is just a rough estimation. A properly designed prospective study might reveal a considerable shorter time to negative PCR as an indicator for successful therapy.

In conclusion, the present study demonstrates that *Strongyloides* PCR is an efficient diagnostic tool for the management of imported strongyloidiasis, with high potential for monitoring treatment efficacy.

## Data availability statement

The original contributions presented in the study are included in the article/**Supplementary Files**, further inquiries can be directed to the corresponding author.

## Ethics statement

Ethical approval was not required for the studies involving humans because the retrospective nature of this study, without any additional intervention or use of biomaterials, no informed consent was obtained. The studies were conducted in accordance with the local legislation and institutional requirements. The human samples used in this study were acquired from a by- product of routine care or industry. Written informed consent to participate in this study was not required from the participants or the participants’ legal guardians/next of kin in accordance with the national legislation and the institutional requirements.

## Author contributions

LW: Writing – original draft, Writing – review & editing, Data curation, Formal Analysis. SVA: Data curation, Formal Analysis, Writing – original draft, Writing – review & editing. LVL: Writing – original draft, Writing – review & editing, Conceptualization. EW: Writing – original draft, Writing – review & editing. JV: Writing – original draft, Writing – review & editing, Conceptualization.
