# Supplementary material for: Real-time PCR for diagnosing and monitoring treatment effect of Strongyloides stercoralis infection in a non-endemic setting
Source: Front Parasitol. 2023 Oct 27;2:1277372. doi: 10.3389/fpara.2023.1277372 (PMC11731781; doi:10.3389/fpara.2023.1277372)
Supplement: Supplementary file 1 [file Table_1.pdf]

Supplementary table, primer and probe sequences for two multiplex helminth PCRs

	Oligo name	Sequence	target	Amplicon	references
**Multiplex 1**					
Trichuris trihiura					
	Tt-F1	TTGAAACGACTTGCTCATCAACTT	SSU	76 bp	(1)
	Tt-R1	CTGATTCTCCGTTAACCGTTGTC	SSU	76 bp	(1)
	Tt-2-YakimaYellowBhq1	CGATGGTACGCTACGTGCTTACCATGG	SSU	76 bp	(1)
Schistosoma					
	Schis-F1	GGTCTAGATGACTTGATYGAGATGCT	ITS2	77 bp	(2)
	Schis-R1	TCCCGAGCGYGTATAATGTCATTA	ITS2	77 bp	(2)
	Schis-1-FAMBhq1	TGGGTTGTGCTCGAGTCGTGGC	ITS2	77 bp	(2)
Strongyloides stercoralis					
	Stro-F1	GAATTCCAAGTAAACGTAAGTCATTAGC	SSU	101 bp	(3, 4)
	Stro-R1	TGCCTCTGGATATTGCTCAGTTC	SSU	101 bp	(3, 4)
	Stro-4-TRBhq2	ACACACCGSCCGTCGCTGC	SSU	101 bp	(3, 4)
Taenia solium					
	Tsol_ITS_145F	ATGGATCAATCTGGGTGGAGTT	ITS1	86 bp	(5)
	Tsol_ITS_230R	ATCGCAGGGTAAGAAAAGAAGGT	ITS1	86 bp	(5)
	Tsol_ITS_169Tq_Q705Bhq2	TGGTACTGCTGTGGCGGCGG	ITS1	86 bp	(5)
Taenia saginata					
	Tsag_ITS_F529	GCGTCGTCTTTGCGTTACAC	ITS1	79 bp	(5)
	Tsag_ITS_R607	TGACACAACCGCGCTCTG	ITS1	79 bp	(5)
	Tsag_ITS_581Tq_Q705Bhq2	CCACAGCACCAGCGACAGCAGCAA	ITS1	79 bp	(5)
**Multiplex 2**					
Ancylostoma					
	Anc-F1	GAATGACAGCAAACTCGTTGTTG	ITS2	71 bp	(6)
	Anc-R1	ATACTAGCCACTGCCGAAACGT	ITS2	71 bp	(6)
	Anc-2-FAM MGB	ATCGTTTACCGACTTTAG	ITS2	71 bp	(6)
Necator americanus					
	Nec-F1	CTGTTTGTCGAACGGTACTTGC	ITS2	101-104 bp	(6)
	Nec-R1	ATAACAGCGTGCACATGTTGC	ITS2	101-104 bp	(6)
	Nec-2-FAM MGB	CTGTACTACGCATTGTATAC	ITS2	101-104 bp	(6)
	Nec-2-FAM MGB	CTGTACTACGCATTGTATGT	ITS2	101-104 bp	(4)
Ascaris					
	Alum-F1	GCCACATAGTAAATTGCACACAAAT	ITS1	134 bp	(7)
	Alum-R1	GCCTTTCTAACAAGCCCAACAT	ITS1	134 bp	(7)
	Alum-2_YakimaYellowBhq1	TTGGCGGACAATTGCATGCGAT	ITS1	134 bp	(8)

Hymenolepis nana					
	HnITS1_1593F	CATTGTGTACCAAATTGATGATGAGTA	ITS1	88 bp	(9)
	HnITS1_1680R	CAACTGACAGCATGTTTCGATATG	ITS1	88 bp	(9)
	Hna_1622T_TexasRedBhq1	CGTGTGCGCCTCTGGCTTACCG	ITS1	88 bp	(9)
Enterobius vermicularis					
	Ev_ITS1_291F	CGGTGTAATTTTGTTGGTGTCTATG	ITS1	143 bp	(9)
	Ev_ITS1_433R	TGGCAGCATTGCAAACTAATG	ITS1	143 bp	(9)
	Ev_ITS1_319_ Q705Bhq2	TGTGCCAGTCAACGCCTAAACCGTC	ITS1	143 bp	(9)

SSU Small subunit (18S)
ITS1 Internal transcribed spacer 1
ITS2 Internal transcribed spacer 2

References

1. Liu J, Gratz J, Amour C, Kibiki G, Becker S, Janaki L, Verweij JJ, Taniuchi M, Sobuz SU, Haque R, Haverstick DM, Houpt ER. A Laboratory-Developed TaqMan Array Card for Simultaneous Detection of 19 Enteropathogens. Journal of Clinical Microbiology. 2013;51:472-480. doi:10.1128/JCM.02658-12; 10.1128/JCM.02658-12.

2. Obeng BB, Aryeetey YA, de Dood CJ, Amoah AS, Larbi IA, Deelder AM, Yazdanbakhsh M, Hartgers FC, Boakye DA, Verweij JJ, van Dam GJ, van Lieshout L. Application of a circulating-cathodic-antigen (CCA) strip test and real-time PCR, in comparison with microscopy, for the detection of Schistosoma haematobium in urine samples from Ghana. Annals of Tropical Medicine and Parasitology. 2008;102:625-633. doi:10.1179/136485908X337490; 10.1179/136485908X337490.

3. Verweij JJ, Canales M, Polman K, Ziem J, Brienen EA, Polderman AM, van Lieshout L. Molecular diagnosis of Strongyloides stercoralis in faecal samples using real-time PCR. Transactions of the Royal Society of Tropical Medicine and Hygiene. 2009;103:342-346. doi:10.1016/j.trstmh.2008.12.001; 10.1016/j.trstmh.2008.12.001.

4. Verweij JJ. Validation and maintaining laboratory developed molecular tests compliant with ISO15189 for diagnosis of intestinal parasitic infections. Expert Rev Mol Diagn. 2022 Jun;22(6):595-601. eng. Epub 20210902. doi:10.1080/14737159.2021.1971974. Cited in: Pubmed; PMID 34424112.

5. Praet N, Verweij JJ, Mwape KE, Phiri IK, Muma JB, Zulu G, van Lieshout L, Rodriguez-Hidalgo R, Benitez-Ortiz W, Dorny P, Gabriël S. Bayesian modelling to estimate the test characteristics of coprology, coproantigen ELISA and a novel real-time PCR for the diagnosis of taeniasis. Tropical Medicine & International Health. 2013;18(5):608-14:608-614. doi:10.1111/tmi.12089.

6. Verweij JJ, Brienen EA, Ziem J, Yelifari L, Polderman AM, Van Lieshout L. Simultaneous detection and quantification of Ancylostoma duodenale, Necator americanus, and Oesophagostomum bifurcum in fecal samples using multiplex real-time PCR. The American Journal of Tropical Medicine and Hygiene. 2007;77:685-690.

7. Liu J, Gratz J, Amour C, Nshama R, Walongo T, Maro A, Mduma E, Platts-Mills J, Boisen N, Nataro J, Haverstick DM, Kabir F, Lertsethtakarn P, Silapong S, Jeamwattanalert P, Bodhidatta L, Mason C, Begum S, Haque R, Praharaj I, Kang G, Houpt ER. Optimization of Quantitative PCR Methods for Enteropathogen Detection. PloS one. 2016. p. e0158199.

8. Wiria AE, Prasetyani MA, Hamid F, Wammes LJ, Lell B, Ariawan I, Uh HW, Wibowo H, Djuardi Y, Wahyuni S, Sutanto I, May L, Luty AJ, Verweij JJ, Sartono E, Yazdanbakhsh M, Supali T. Does treatment of intestinal helminth infections influence malaria? Background and methodology of a longitudinal study of clinical, parasitological and immunological parameters in Nangapanda, Flores, Indonesia (ImmunoSPIN Study). BMC Infectious Diseases. 2010;10:77. doi:10.1186/1471-2334-10-77; 10.1186/1471-2334-10-77.

9. Koller T, Hahn A, Altangerel E, Verweij JJ, Landt O, Kann S, Dekker D, May J, Loderstadt U, Podbielski A, Frickmann H. Comparison of commercial and in-house real-time PCR platforms for 15 parasites and microsporidia in human stool samples without a gold standard. Acta Tropica. 2020;207:105516. doi:S0001-706X(19)31769-3 [pii].
